# Structure-Function Guided Extraction and Scale-Up of Pea Protein Isolate Production

**DOI:** 10.3390/foods11233773

**Published:** 2022-11-23

**Authors:** Lucy Hansen, Fan Bu, Baraem P. Ismail

**Affiliations:** Food Science and Nutrition Department, University of Minnesota, 1334 Eckles Ave., Saint Paul, MN 55108, USA

**Keywords:** pea protein isolate, salt extraction, pH extraction, ultrafiltration, benchtop versus pilot plant production, protein structure and functionality

## Abstract

The lack of adequate guidance and control of the extraction conditions as well as the gap between bench- and industrial-scale production, contributes to the poor functionality of commercial pea protein isolate (cPPI). Therefore, pea protein extraction conditions were evaluated and scaled up to maximize protein purity and yield, while maintaining structural integrity, following mild alkaline solubilization with isoelectric precipitation and salt solubilization coupled with membrane filtration. Both extraction methods resulted in high protein yield (>64%) and purity (>87%). Structure-function characterization illustrated the preserved structural integrity of PPI samples and their superior solubility, gelation, and emulsification properties compared to cPPI. Results confirmed, for the first time, that double solubilization at mild pH (7.5) can replace single solubilization at high alkalinity and achieve a similar yield while preserving structural integrity. Additionally, this study demonstrated, the scalability of the benchtop salt extraction coupled with ultrafiltration/diafiltration. Scaling up the production eliminated some structural and functional differences between the salt-extracted PPI and pH-extracted PPI. Scaling-up under mild and controlled conditions resulted in partial denaturation and a low degree of polymerization, coupled with the superior functionality of the produced isolates compared to cPPI. Results of this work can be used as a benchmark to guide the industrial production of functional pea protein ingredients.

## 1. Introduction

Produced from yellow field peas (*Pisum sativum* L.), pea protein has similar profile and nutritional quality compared to soy protein [1,2]. Therefore, pea protein ingredients are being incorporated into several food and beverage applications, replacing soy and animal-based protein ingredients. However, the functional properties of pea protein namely solubility, gelation, and emulsification are inferior to that of soy protein [3], hindering its expanded use. The food industry, accordingly, is searching for effective measures to enhance the functionality of pea protein for successful incorporation in various applications.

While soy protein ingredients have gone through years of research and development to improve functionality through processing and breeding, pea protein ingredient development is yet to catch up. The protein in commercially available pea protein isolates (PPI) is generally completely denatured, extensively polymerized, and has a relatively high surface hydrophobicity [4]. These structural characteristics contribute to challenging functionality [4]. Preserving the protein structural integrity during the production of PPI may contribute to desired functional attributes and better inclusion prospects in different applications.

The functionality of pea protein is not only affected by the inherent protein profile, but also by the structural changes that take place under certain extraction and processing conditions during the protein isolation process. Commercial PPI is mostly produced by pH extraction, utilizing alkaline solubilization of the protein to separate it from starch and fiber, followed by precipitation at the protein’s isoelectric point to remove soluble compounds and enhance the purity [5,6,7,8]. Differences in pH treatment, holding time, number of solubilizations, heat treatments, and drying conditions, contribute to differences in the protein profile and structure, ultimately impacting functionality. Several studies investigated pea protein extractions at pH levels above pH 8 [7,9,10,11] and the impact on protein yield; however, high alkalinity coupled with adverse heat treatment during production cause protein denaturation and subsequent polymerization, thus negatively impacting functionality.

Protein isolates can also be produced by salt extraction, utilizing a salt solubilization step followed by “salting out” to concentrate the protein. However, this extraction process results in high waste streams and salty protein isolates that require several washes and diafiltration [12]. A few studies investigated salt extraction with “salting out” step for the production of protein isolate [13,14,15]. However, such isolation method is currently not adapted in industry due to the high waste stream, high use of water, and low efficiency. Alternative concentration techniques such as membrane filtration may replace the “salting out” step, which could make this extraction method more feasible. There is limited research on the optimization of salt extraction conditions, including salt concentration and type of membrane filtration, for optimal pea protein purity and yield [10,16,17,18,19]. A few of these studies were based on benchtop trials following micellar precipitation and/or using dialysis membranes [11,17], while only one study was based on pilot plant trials using ultrafiltration/diafiltration membranes [18,19,20]. However, there are no reported comparative studies to determine scalability of a benchtop salt extraction process involving membrane purification. Additionally, there are limited reports on the structural and functional properties of pea protein as impacted by pH extraction versus salt extraction under industry feasible conditions [10,19].

Protein extraction conditions should be selected and controlled based on their efficiency in extracting protein, while maintaining structural integrity. Extraction efficiency can be determined by tracking protein purity and yield. Mild and feasible extraction conditions that produce PPI of high protein purity and yield and acceptable functionality need to be explored. Therefore, the objectives of this work were: (1) evaluate pea protein extraction conditions to maximize protein purity and yield following mild alkaline solubilization with isoelectric precipitation and salt solubilization coupled with membrane filtration; (2) characterize the impact of the two extraction methods on the protein structure and functionality; (3) and for the first time, translate controlled benchtop extraction conditions to pilot scale production of PPI, while evaluating the impact of scaling on the structure and functionality of the protein.

## 2. Materials and Methods

### 2.1. Materials

Yellow field pea flour (~20% protein content) was provided by AGT Foods (Regina, SK, Canada) and commercial pea protein isolate (cPPI, 81.2% protein, 3.86% ash), PURIS™ Pea Protein, was provided by Puris Foods (Minneapolis, MN, USA). Commercial whey protein isolate (cWPI, 94.6% protein, 4.10% ash), BiPro^®^, was provided by Agropur Ingredients (Eden Prairie, MN, USA). Defatted soy flour (~53% protein) and commercial soy protein isolate (cSPI, ~90.7% protein), ProFam^®^ 974, were provided by Archer Daniels Midland (ADM) (Decatur, IL, USA). All aforementioned samples were stored at −20 °C prior to usage. SnakeSkin™ dialysis tubing (3.5 kDa cut off) and Sudan Red 7B were purchased from Thermo Fisher Scientific™ (Waltham, MA, USA). Criterion™ TGX™ 4–20% precast gels, Laemmli 4X loading buffer, Imperial™ Protein Stain, 10X Tris/Glycine/SDS running buffer and Precision Plus molecular weight marker were purchased from Bio-Rad Laboratories, Inc. (Hercules, CA, USA). Vivaflow^®^ membrane ultrafiltration crossflow cassettes (3 kDa MWCO) were purchased from Sartorius™ (Gottingen, Germany). All other chemical reagents and supplies were purchased from Thermo Fisher Scientific and Sigma-Aldrich.

### 2.2. Benchtop Pea Protein Extractions

#### 2.2.1. PPI Production Following pH Extraction

Solubilization pH, precipitation pH, solubilization time, number of solubilizations, and use of dialysis were evaluated for high protein purity and yield. In triplicate, pea flour was suspended in double deionized water (DDW, 1:10 *w*/*v*) and adjusted to pH 7, 7.5 or 8 with 2N NaOH. The suspensions were stirred for 1 h or 2 h then centrifugated at 5000× *g* for 30 min, and the supernatant was collected. The residual pellet was either lyophilized directly or was redispersed in DDW for another solubilization cycle. The supernatant from the second solubilization was combined with the first supernatant. The pH of the supernatant (or combined supernatants) was adjusted to the isoelectric point (pH 4.5 or 5) and centrifuged at 5000× *g* for 10 min. The precipitate was then redispersed in DDW (1:4 *w*/*v*) and the pH was adjusted to 7 prior to lyophilization. Fractions obtained from every step (the pellet from the first centrifugation step, the supernatant from the second centrifugation step, and the final neutralized PPI fraction) were lyophilized, weighed, and analyzed for protein content following a Dumas method (AOAC 990.03) using a LECO^®^ FP828 nitrogen analyzer (LECO, St. Joseph, MI, USA). Mass balance was tracked and used to determine the final protein yield. The effect of dialysis was also investigated, where the neutralized PPI fraction was either lyophilized as is or was dialyzed at 4 °C against DDW using a 3.5-kDa cutoff membrane. Ash content (AOAC method 942.05) was determined to evaluate the efficiency of dialysis. When not in use, the samples were stored at −20 °C.

#### 2.2.2. PPI Production Following a Salt Solubilization Coupled with Membrane Filtration Method

Pea protein extraction pretrials using salt solubilization coupled with membrane filtration (salt extraction) were performed to select the salt concentration. Selected salt concentration was based on extraction yields. The lowest salt concentration (0.5 M) that produced over 80% protein yield was chosen. The conditions for membrane filtration and protein purification were then evaluated by comparing the effects of membrane ultrafiltration (UF), dialysis, or UF followed by dialysis. The use of membrane filtration and/or dialysis was investigated as an alternative way to “salting out” for the concentration and purification of proteins solubilized in dilute a salt solution. To produce salt extracted PPI, pea flour, in triplicate, was solubilized in 0.5 M NaCl (1:20 *w*/*v*) for 1 h at the natural pH (6.4) and at room temperature (23 °C). The suspension was then centrifuged at 5000× *g* for 30 min to separate insoluble starch and fiber. The supernatant was neutralized and subjected to tangential ultrafiltration (UF), dialysis, or UF coupled dialysis to assess the efficiency of salt reduction. For ultrafiltration, the benchtop Sartorius Vivaflow^®^ 200 system was used with two Vivaflow^®^ membrane (3 kDa MWCO) cassettes running in parallel to increase the speed of filtration. The system was set up according to manufacturer instructions, with the protein solution in a feed reservoir and the feed tube connected to a peristaltic pump (Masterflex Easy Load Pump Head- Size 15, Masterflex Economy Drive Peristaltic Pump 230 V, Sartorius) to pump the feed solution under pressure (2.5 bars) across the membranes, to concentrate the samples down to 50 mL, followed by diafilteration using 6 volumes of DDW (300 mL total) to continually decrease the concentration of salt and other low molecular weight (MW) compounds. After diafiltration, the solution was further concentrated to 25 mL. After filtration and/or dialysis, the protein solution was lyophilized and stored at −20 °C before subsequent analysis. The protein purity, protein yield, and ash content of PPI were determined following the abovementioned methods and used to determine the favorable extraction conditions.

### 2.3. Scaled-Up Pea Protein Extractions in the Pilot Plant

The selected pH and salt extraction methods were scaled up in the Joseph J. Warthesen Food Processing Center at the University of Minnesota to determine how the benchtop extractions translated to larger scale production. The ultimate goal of scaling up pea protein extraction under controlled conditions is to produce functional PPI that can be mass produced by the food industry for widespread use. Therefore, it was important to examine how well the selected benchtop extraction conditions translate to a larger scale. For both the pH- and salt-extractions, there were some unavoidable differences between the benchtop and scaled-up (SU) extractions. Differences were encountered when benchtop equipment did not have a direct analog in the pilot plant. First, the centrifuges used differed in their separation power. The benchtop Beckman floor centrifuge forms dry, compact pellets and a clear supernatant, while the horizontal decanter centrifuge (Westfalia Separator AG, 3.8 L/min, GEA Westfalia Separator Group Gmbh, Oelde, Germany) in the pilot plant cannot achieve the same level of separation. To help improve separation, a desludging disc centrifuge (Westfalia SB7, 3.8 L/min, GEA Westfalia Separator Group Gmbh, Oelde, Germany) was used in sequence to clarify the supernatant further. Additionally, large scale dialysis is infeasible, so ultrafiltration/diafiltration (UF/RO unit, 15–20 psi inlet, 10–15 psi outlet, PTI Advanced Filtration, PTI Technologies, St. Louis, MO, USA with tangential/cross flow and a spiral wound membrane, 3 kDa MWCO) was used instead. Total solids of the permeate was constantly measured using a CEM AVC-80 Microwave Moisture/Solids Balance Analyzer (CEM, Charlotte, NC, USA) to monitor salt removal. Furthermore, ingredients produced in the pilot plant must be food-grade, so the PPI was pasteurized after filtration. Following pasteurization, the PPI was homogenized to help improve ease of drying. Lyophilizing is not commonly done in industry because it is time consuming and costly, so the scaled-up PPI samples were dried using a SPX Flow Anhydro Spray Dryer (9.5% TS, 180 °C inlet, 90 °C outlet, 9 L/h) with a wheel type atomizer (24,500 rpm) (SPX Flow Inc., Charlotte, NC, USA). Lastly, extractions in the pilot plant spanned across two days of processing, so precautionary steps were added to the extraction methods to prevent microbial growth overnight.

#### 2.3.1. Scaled-Up pH-Extraction

Pea flour was dispersed in deionized (DI) water (1:10, *w*/*v*; pH 7.5) and agitated in a jacketed tank (568 L) with automated stirrer for 1 h at room temperature (23 °C). The solution was then separated using a horizontal decanter centrifuge and clarified with a desludging disc centrifuge. The separated liquid was set aside in the cold room (6–8 °C). The precipitate was weighed, and the total solids (%TS) was measured. The pellet was resuspended at 1:10 *w/v* and the pH adjusted to 7.5, followed by agitation in the jacketed tank for another hour. The solution was then passed through the decanter centrifuge and desludging centrifuge again, and the separated liquid was collected and combined with the separated liquid from earlier in a clean jacketed tank. The combined solution was then adjusted to pH 4.5 and agitated for 10 min. The solution was then passed through the decanter centrifuge, and the proteinaceous precipitate was collected. The supernatant was pumped through the desludging centrifuge to ensure that no additional solids precipitated. The combined solids were transferred to a jacketed tank and reconstituted in DI water at 1:4 *w*/*v*. The solution was adjusted to pH 3 and left in the cold room (6–8 °C) overnight. Leaving the protein at its isoelectric point overnight would cause protein denaturation, leading to aggregation and irreversible polymerization, while neutralizing the supernatant could allow for microbial growth overnight. At the beginning of the second day, the solution was neutralized and agitated for 1 h at room temperature (23 °C). Ultrafiltration/diafiltration was performed in place of dialysis using a UF/RO unit described above. The protein solution was diafiltered with a set up similar to the benchtop diafiltration. DI water was added (40 L at a time) until the % TS of the exiting permeate stream was 0.00%. Following ultrafiltration/diafiltration, the retentate was pasteurized by running the solution through a high temperature short time (HTST; 73 °C for 15 s) processing system (MicroThermics^®^ Electric Model 25HV Hybrid, 57–170 L-/h, MicroThermics^®^ Inc., Raleigh, NC, USA), followed by two-stage homogenization (Gaulin 125 L, 2500 psi, 227 L/h, Manton-Gaulin Mfg. Co. Inc., Everett, MA, USA). The solution was then spray dried using a SPX Flow Anhydro Spray Dryer described above. Protein purity and ash content of the final SU-pH PPI were determined by Dumas and dry ashing, respectively, to assess how the pilot plant extraction compared to the benchtop extraction. When not in use, the sample was stored at −20 °C.

#### 2.3.2. Scaled-Up Salt Extraction

Pea flour was dispersed in 0.5 M NaCl (1:20 *w*/*v*) at its natural pH (pH 6.4) and agitated in a jacketed tank (568 L) with automated stirrer for 1 h at room temperature (23 °C). The solution was then separated using a horizontal decanter centrifuge and clarified with a desludging centrifuge. The separated liquid was set aside in the cold room (6–8 °C). The precipitate was weighed, and the total solids (%TS) was measured. The pellet was resuspended in 0.5 M NaCl at 1:5 *w/v* and agitated in a jacketed tank for 30 min. The solution was then passed through the decanter centrifuge and desludging centrifuge again, and the separated liquid was collected and combined with the separated liquid from earlier in a jacketed tank. The solution was neutralized and left stirring in the cold room (6–8 °C) overnight. The salt content of the solution and the cool temperature were assumed to be sufficient to prevent microbial growth. The following day, the protein solution was ultrafiltered/diafiltered pasteurized, homogenized, and spray dried as described above. It is worth noting that a membrane MWCO of 3 kDa was selected to avoid loss of small molecular weight proteins and peptides in contrast to 10, 20, and 50 kDa MWCO that have been used previously [18,19,20]. Additionally, when scaling, it was essential to simulate the benchtop filtration setup. Protein purity and ash content of the final SU-salt PPI were determined by Dumas and dry ashing, respectively, to assess the scalability of salt extraction. When not in use, the sample was stored at −20 °C.

### 2.4. Structural Characterization

#### 2.4.1. Protein Profiling by SDS-PAGE and SE-HPLC

The protein subunit distribution in the different PPI samples and references was visualized using SDS polyacrylamide gel electrophoresis (SDS-PAGE) as previously described by Laemmli [21] and modified by Bu [4]. Sample aliquots (5 µL; containing ~50 µg protein) and MW standard (10 µL) were loaded onto a 4–20% acrylamide gel under non-reducing and reducing conditions. The gel was electrophoresed, stained, de-stained, and imaged as reported previously by Boyle [22].

Size exclusion high performance liquid chromatography (SE-HPLC), as previously described by Bu [4], was used to evaluate the molecular weight distribution of the proteins in the different samples. Pea protein (0.1 g) was solubilized in 10 mL of 0.05 M sodium phosphate with 0.1 M sodium chloride buffer at pH 7 for two hours, after which the samples were filtered through a 0.45 µm filter. The same Shimadzu system, analytical column, MW calibration standards, chromatographic method, and identification process reported by Bu [4] were used to obtain the molecular weight distribution.

#### 2.4.2. Protein Denaturation as Determined by Differential Scanning Calorimetry (DSC)

The denaturation temperature and enthalpy of the different protein samples were determined using DSC (Mettler Toledo, Columbus, OH, USA) equipped with the STARe software, version 11, as described by Boyle [22] and modified by Bu [4].

#### 2.4.3. Measurement of Zeta Potential and Surface Hydrophobicity

A Zetasizer Nano Z instrument (Malvern Panalytical, Malvern, UK), equipped with Malvern’s Zetasizer software (version 7.13), was used to determine the zeta potential of the protein samples as described previously Bu [4]. A fluorescence-based method previously reported by Boyle [22], without modification, was used to determine the surface hydrophobicity of the protein samples.

### 2.5. Functional Properties

The solubility of the protein samples (5% protein concentration, *w*/*v*) was determined at pH 3.4 and 7 with and without heating at 80 °C for 30 min following the procedure outlined by Wang [23], without modification. Solubility was expressed as the percentage of protein in the supernatant to the total protein in the initial solution as determined by the Dumas method.

Thermally induced gels were prepared by heating protein solutions (at 15% or 20% protein *w*/*v*) at 95 °C for 10 min or 20 min. The samples were then cooled to room temperature and the gel strength was determined using a TA-TX Plus texture Analyzer (Stable Micro Systems LTD, Surrey, UK) with a 100 mm diameter probe, at a test speed of 1 mm s^-1^ and distance of 0.5 mm from the plate. The force (N) required to rupture the gel was reported as gel strength.

Emulsification capacity (EC, at 1% or 2% protein in DDW, *w*/*v*), activity index (EAI), and stability (ES) of the different protein samples at pH 7 were determined following the methods outlined by Boyle [22] and modified by Hinnenkamp [24].

### 2.6. Statistical Analysis

All measurements were performed in triplicate, and analysis of variance (ANOVA) was performed using RStudio software version 1.1.463 for Mac (Rstudio, Inc., Boston, MA, USA). When determining significant difference between two means, a student’s unpaired t-test was used (*p* ≤ 0.05). For determining significant differences among three or more means, Tukey–Kramer Honest Significant Difference (HSD) multiple means comparison test was (*p* ≤ 0.05).

## 3. Results and Discussion

### 3.1. Effect of Different pH Extraction Conditions on the Efficiency of PPI Production

Solubilization pH, isoelectric precipitation pH, solubilization duration, number of solubilizations, and use of dialysis were each evaluated, in turn. With all other conditions kept constant, the solubilization pH was set at either pH 7, 7.5, or 8. Compared to PPI samples extracted at pH 7.5 and 8, PPI extracted at pH 7 had the lowest protein yield with significantly higher protein content and % residual protein in the discarded pellet fraction (Table 1), demonstrating that pH 7 was least effective in extracting proteins from pea flour. Solubilizing the protein at pH 8, on the other hand, resulted in significantly higher PPI protein yield compared to PPI samples extracted at pH 7 or 7.5. While it is known that plant proteins have increased solubility at elevated pH levels [25], high pH can coextract other non-protein constituents such as polysaccharides [26,27], and can negatively impact the functionality of the protein due to inducing protein denaturation and subsequent aggregation [28]. Additionally alkaline pH promotes oxidation that may lead to browning, off-flavor, and further protein polymerization [29,30,31,32,33]. Therefore, pH 7.5 was selected as the solubilization pH, as it resulted in a comparable and acceptable PPI protein purity (>85% protein) and yield.

Next, the isoelectric precipitation pH was evaluated. Pea protein, similar to soy protein, has low solubility over a wide range of acidic pH 3.5–5.5 [34,35], with isoelectric precipitation commonly performed at pH 4.5 or 5 [35,36]. A comparative testing was performed to determine the direct impact, if any, of the precipitation pH on the protein yield. Though there were no significant differences in the final PPI protein purity or yield, the supernatant fraction that was discarded after protein precipitation at pH 5 had a significantly higher protein content and % protein lost than the supernatant discarded after protein precipitation at pH 4.5 (Table 1). Therefore, pH 4.5 was selected for protein precipitation.

With regard to solubilization duration, there was no significant differences in protein yield and purity when solubilized for one hour versus two (Table 1). Therefore, one hour of solubilization was chosen for industrial efficiency. Similarly, Hoang [25] did not observe significant increase in pea protein extraction yield with increasing solubilization duration from 15 to 45 min. Pea flour, however, was solubilized at pH 10, where the proteins are more readily soluble than at pH 7.5, the chosen pH for the present study. It is assumed that some protein was retained with the pellet post solubilization at pH 7.5. Therefore, additional round of solubilization with fresh solvent was tested next.

Double solubilization, for 1 h each, was indeed effective in extracting additional protein from the first pellet, which ultimately led to ~12% increase in protein yield (58.1 vs. 69.9%), accompanied by a significant reduction (10%) in % residual protein in the discarded pellet fraction (Table 1). The reported PPI protein yield obtained from alkaline solubilization with isoelectric precipitation is around 60–70%, mainly achieved by solubilizing proteins at extreme alkaline conditions (pH 8–10) [17]. In contrast, the relatively similar or superior yield observed in this study when employing double solubilization was achieved using mild alkaline solubilization pH, hence preserving the protein structure. This is the first study to discover and illustrate that double solubilization at mild pH can replace single solubilization at high alkalinity and achieve similar yield, while potentially preserving structural integrity.

The effect of dialysis was investigated next to determine the impact of desalting and removal of small molecules on the purity and yield. PPI with high protein purity is desirable from a nutritional standpoint and may demonstrate superior functional properties due to the reduced content of other interfering components. Dialysis, however, caused a 5% decrease in the protein yield (Table 1), attributed to an additional transfer step and incomplete recovery of the protein from the dialysis tubing, and did not successfully decrease the ash content. While the difference in yield is statistically significant, the yield remains relatively high. Reducing the salt content using ultrafiltration/diafiltration is more effective compared to dialysis; therefore, it was decided to pursue membrane filtration during scale up trials to enhance upon the protein functionality even though the yield might be slightly compromised.

Based on these findings, the final extraction conditions were double solubilization of pea flour for one hour at pH 7.5, protein precipitation at pH 4.5, and the use of filtration. These nondenaturing extraction conditions were chosen for the scaled-up production of pH extracted PPI.

### 3.2. Effect of Membrane Filtration on the Production Efficiency of Salt Extracted PPI

Salt extraction of plant proteins is less researched than alkaline extraction and is not currently used in industry for the production of PPI. Studies investigating salt extraction mostly recover the PPI by “salting out”, which is not industry feasible due the use of excessive salt and water. Other studies report micellar precipitation (also called “hydrophobic out”), in which the proteinaceous supernatant from “salting in” gets diluted with cold water, leading to increased hydrophobic interactions causing protein precipitation and reduced protein solubility [10,12,17,37].

In this study, membrane filtration, in place of “salting out”, was used to purify the protein after salt solubilization. For the first time, the utilization of membrane ultrafiltration (UF) compared to dialysis, separately, and in combination, were evaluated in terms of protein purity, yield, and ash content. The initial salt solubilization step was the same for all three purification treatments, thus both the protein purity and % protein residue of the discarded pellet were not impacted (Table 1). Ultrafiltration alone, using the benchtop membrane system, resulted in the lowest protein purity and highest ash content, demonstrating that it was not sufficient to completely remove all salt from the proteinaceous supernatant. Dialysis was significantly more effective in increasing protein purity and decreasing ash content, though the ash content was still relatively high, indicating that not all salt was removed (Table 1). The combination of UF and dialysis had comparative high yield and had significantly the highest PPI protein purity and the lowest ash content of the three purification treatments (Table 1), thus was selected as the purification treatment. This outcome could translate to successful use of UF coupled with diafiltration on a pilot or industrial scale.

To the best of our knowledge, very limited work has been done on the use of solubilization in dilute NaCl, followed by UF, to produce PPI. While ultrafiltration has been used following an alkaline or acidic solubilization step, or as further purification after isoelectric precipitation [11,16], it is not well characterized as a protein concentration step following salt solubilization. Tian [19] and Taherian [20] reported the use of ultrafiltration post salt solubilization of pea protein, however, 20 and 50 kDa MWCO membrane were used, respectively, which could potentially lead to loss of a considerable amount of relatively small molecular weight proteins.

Scalability of UF coupled with diafiltration could potentially be considered costly to operate. However, membrane filtration is commonly utilized in the dairy industry to produce whey protein concentrates and isolates of high quality and value. Additionally, in an economic evaluation study comparing alkaline extraction with isoelectric precipitation to membrane isolation for the production of soy protein isolate, there were no significant differences between in the cost of production [38]. Therefore, it is essential to investigate the potential differences between benchtop extractions and scaled-up extractions, under controlled conditions with small MWCO (3 kDa), to determine the feasibility of such extraction process and its impact on protein profile, structure, and functionality in comparison to the conventional pH extraction protocol.

### 3.3. Pilot Production of PPI Following the Selected Extraction/Purification Conditions

Pilot scale production of PPI was performed closely following the selected benchtop extraction/purification conditions, with slight modifications to accommodate industrial practices, including the use of diafiltration instead of dialysis, pasteurization for safety measures, and spray drying instead lyophilization. The SU production achieved similar PPI purity to the benchtop counterpart. SU-pH PPI had a protein purity of 88.7%, while SU-salt PPI had a protein purity of 92.4%. Ash content of the SU-salt PPI (1.66%) was comparable to that of the benchtop salt-PPI (1.56%), while ash content of the SU-pH PPI (2.94%) was significantly (*p* < 0.05) lower than the benchtop pH-PPI (4.96%). In their study on pilot scale production of salt extracted PPI, Tian [19] reported a lower PPI protein purity (81.1%), which could be attributed to higher retention of starch and fiber potentially due to less ideal separation mechanism, coupled with probable loss of protein when using high MWCO membrane (20 kDa). While there are a couple of reports on scaling up of salt extracted PPI, the literature still lacks a thorough and comparative investigation of the impact of different extraction methods, performed under mild conditions, and their scaling up, on the structural changes and consequent functionality compared to commercially available PPI.

### 3.4. Effect of Extraction Conditions and Scaling up on Protein Profile

**Figure 1:**
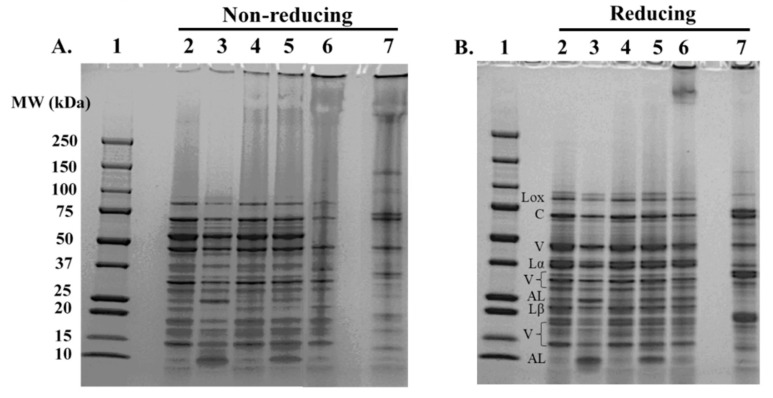
SDS-PAGE gel visualization of the protein profiles of protein isolate samples under non-reducing (**A**) and reducing (**B**) conditions. Lane 1: molecular weight (MW) marker; Lane 2: pH-PPI; Lane 3: salt-PPI; Lane 4: SU-pH PPI; Lane 5: SU-salt PPI; Lane 6: cPPI; Lane 7: cSPI. Lox: lipoxygenase; C: convicilin; V: vicilin; Lα: acidic subunit of legumin; Lβ: basic subunit of legumin; AL: albumin.

Protein bands corresponding to lipoxygenase (~94 kDa), convicilin (~72 kDa), vicilin (~50 kDa), and legumin (~60 kDa) (Figure 1A) were apparent in all extracted PPIs, similar to previous reports [29,39,40]. Since convicilin and vicilin do not contain disulfide linkages, their corresponding bands were similar under reducing and nonreducing conditions (Figure 1A,B). On the other hand, legumin was cleaved into its acidic (~40 kDa) and basic (~20 kDa) subunits under reducing conditions (Figure 1B).

While benchtop extracted PPI and corresponding SU PPI samples had similar protein bands, there were a few notable differences. The most notable difference was between the benchtop salt-PPI and SU-salt PPI under nonreducing conditions. The upper part of the lane for SU-salt PPI had longitudinal smearing compared to that of the benchtop salt-PPI, indicating the presence of large protein aggregates (Figure 1A, lanes 3&5). Both SU-pH PPI and pH-PPI had smearing in the upper region of their respective lanes under nonreducing conditions (Figure 1B, lanes 2&4), indicating the presence of protein aggregates. However, the smearing was more intense for the SU-pH PPI, with a much darker band at the very top of the lane compared to the benchtop pH-PPI. The smearing and the intensity of the high molecular weight bands at the top of the lanes in the SU PPI samples were markedly less visible under reducing conditions (Figure 1B, lanes 4&5), indicating that these aggregates were formed primarily through disulfide linkages due to the thermal treatment during pilot plant production (pasteurization and spray drying).

When comparing salt-extracted PPI to pH-extracted PPI, unique bands around 25 and 9 kDa were present in salt PPI and SU-salt PPI and absent or less intense in the pH PPI and SU-pH PPI, under both reducing and nonreducing conditions (Figure 1A,B, compare lanes 3&5 to lanes 2&4). This observation indicated that these 25 and 9 kDa proteins have an isoelectric point further from pH 4.5, allowing them to remain soluble during the isoelectric precipitation step of the pH extraction, and hence are lost in the discarded supernatant. These proteins are likely albumins, which have an isoelectric point of around pH 5.5–6 [41,42,43]. Albumins have different structural properties than globulins, so their presence in the salt-extracted PPIs may cause some differences in functionality.

Both commercial references, cPPI and cSPI had intense smearing and high molecular weight bands compared to the benchtop and SU PPI samples. Additionally, the 60 kDa band corresponding to legumin and glycinin in cPPI and cSPI, respectively, was largely absent under nonreducing conditions (Figure 1A, lanes 6&7). However, under reducing conditions, the corresponding acidic and basic subunits around 40 and 20 kDa, respectively, were present in both lanes. Additionally, the smearing in the upper region of the lanes was markedly reduced. The reduction in smearing coupled with the appearance of the acidic and basic subunits indicated that the excessive polymerization, involving the legumin in cPPI and glycinin in cSPI, occurred mostly via disulfide linkages. These observations were similar to those reported previously [40,44], when comparing native PPI and isolated legumin fraction to excessively heated samples.

High molecular weight bands (>250 kDa) were still apparent under reducing conditions, in both cPPI and cSPI. The persistent presence of these large molecular weight bands indicated that the polymerization in these samples involved not only disulfide linkages, but also other covalent linkages [45,46,47]. Therefore, it was concluded that cPPI and cSPI were most likely subjected to high alkalinity and excessive heat treatment during processing, which could impair their functional properties. However, it was noted that under reducing conditions, the bands corresponding to glycinin subunits in cSPI had a relatively higher intensity than legumin counterparts in all PPI samples, indicating that the legumin to vicilin ratio is higher in SPI than in PPI, likely contributing to better functionality of cSPI compared to cPPI, as will be discussed in later sections.

**Figure 2:**
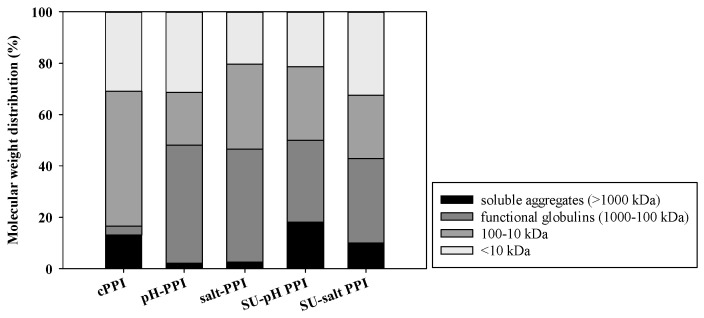
Percent relative abundance of different protein fractions in cPPI reference and PPI samples produced at bench scale and pilot scale. Samples were dissolved in pH 7 phosphate buffer and an-alyzed by SE-HPLC. Bars distribution represents means of *n* = 3.

The protein molecular weight distribution and the relative abundance of major protein fractions in the different isolates were determined using SE-HPLC (Figure 2 and Table 2). Compared to benchtop PPI samples, SU PPI samples had significantly higher relative abundance of soluble aggregates, regardless of extraction type. This observation is likely attributed to the additional processing that the SU samples were subjected to (pasteurization, homogenization and spray drying). Bu [4] reported enhanced functionality upon the formation of soluble aggregates. Therefore, the presence of soluble aggregates in SU PPI might contributed to better functionality compared to benchtop PPI. While cPPI also showed relatively high abundance of soluble aggregates, it had the least relative abundance of functional proteins across all PPI samples (Figure 2), confirming the SDS-PAGE observation (Figure 1A). Proteins in cPPI not only formed soluble aggregates, but they formed larger insoluble aggregates that were most likely filtered out when the sample was passed through the 0.45 µm filter prior to the SE-HPLC analysis, thus had no apparent chromatographic peak. In contrast, all benchtop and SU PPIs had high relative abundance of functional proteins. High level of insoluble aggregates along with the loss in functional proteins would likely impair the functionality of cPPI, whereas the presence of functional proteins and modest amounts of soluble aggregates in the pH and salt PPI samples extracted under selected conditions could contribute to better functionality.

### 3.5. Protein Denaturation as Impacted by Extraction Method and Scale

The impact of the extraction method (pH- vs. salt- extraction) and scale (benchtop vs. SU) on the protein denaturation temperature and enthalpy was assessed using DSC. The benchtop and SU PPI samples had two endothermic peaks corresponding to vicilin and legumin (Table 3). It is likely that the endothermic peak for convicilin overlapped with that of vicilin, as they are structurally similar. Isolated vicilin and convicilin fractions were reported to have the same denaturation temperature [31]. Moreover, convicilin represents a much smaller portion (4–8%) of the total pea proteins than vicilin (up to 52%), thus the majority of the enthalpy of denaturation for the first peak is attributed to vicilin [39].

The legumin In the salt-PPI had slightly higher enthalpy of denaturation compared to that in the pH-PPI (Table 3). This observation compliments the SDS-PAGE observation, where more smearing in the upper part of the lane was noted for pH-PPI compared to salt-PPI (Figure 1A, lanes 2&3). Both observations indicated that salt-extraction was less denaturing than the pH-extraction. On the other hand, vicilin and legumin in both benchtop PPI samples had higher denaturation enthalpies than those in the SU PPI samples (Table 3). This observation confirmed that the processes during pilot plant production resulted in partial denaturation of the proteins, mostly attributed to the thermal treatment during pasteurization and spray drying.

While the SU PPI samples may have been partially denatured compared to the benchtop PPI samples, both were less denatured than the commercial references (cPPI and cSPI). Neither cPPI nor cSPI showed any endothermic peaks, indicating that the vicilin and legumin proteins were completely denatured. This finding supports the protein profiling observations (Figure 1 and Figure 2, Table 2), which showed that both commercial references had excessive aggregation of denatured proteins, as was also reported by other researchers [4,32,48]. While the extraction and processing conditions of these commercial proteins are not known, it is presumed that the commercial references had undergone harsher extraction and thermal processing conditions compared to the selected SU conditions of this study, causing complete denaturation. Specifically, the difference in the denaturation state between the SU PPI samples and the references could be attributed to higher solubilization pH, overnight storage at the isoelectric point, thermal concentration step, and/or different spray drying conditions. The differences in the degree of denaturation will help explain differences in other structural properties and consequently functionality.

### 3.6. Protein Surface Properties as Impacted by the Extraction Method and Scale

Extraction method did not have a significant impact on surface hydrophobicity, while the extraction scale did (Table 3), confirming that thermal treatment had a major impact on the protein structure. This observation is consistent with the denaturation state of SU PPI samples compared to their benchtop counterparts. Denaturation disrupts electrostatic interactions, hydrogen bonding, and hydrophobic interactions that stabilize the native globular protein structure, causing unfolding and exposure of the hydrophobic core [49]. While cPPI in comparison to SU PPI samples was completely denatured (Table 3), there were no significant differences in surface hydrophobicity among these samples. Legumin protein was excessively polymerized in cPPI (Figure 1, lane 6). Upon complete denaturation, surface hydrophobicity will reach a maximum, and with subsequent and progressive polymerization induced by denaturation, it will be reduced [23]. Denatured proteins are attracted to each other via hydrophobic forces; once in close proximity, hydrophobic interactions occur and facilitate disulfide polymerization [50]. While both the SU PPIs and cPPI had protein aggregates, protein aggregation was more extensive in cPPI (Figure 1). Higher degree of denaturation and polymerization will have a detrimental impact on some functional properties, such as solubility, as will be discussed in later sections. Similarly, cSPI was completely denatured (Table 3), thus had high surface hydrophobicity. SPI, however, is unique in that it has a relatively high surface hydrophobicity along with fairly high surface charge [51]. The balance of surface charge and surface hydrophobicity affects how the protein interacts with its surrounding, thus, it impacts functional behavior such as solubility, gelation, and emulsification, as will be discussed in later sections.

Zeta potential is an indication of surface charge, which influences not only the protein solubility but also its intermolecular interactions. All tested proteins carried a net negative charge at pH 7, which is above their isoelectric point. The benchtop pH-PPI had the highest surface charge among all PPI samples, while the benchtop salt-PPI had the lowest surface charge (Table 3). This observation agrees with previous reports of legume proteins produced following pH extraction versus salt extraction [11]. The observed differences in surface charge may be explained by the protein composition of the pH-PPI compared to that of the salt-PPI. The pH-PPI was purified by isoelectric precipitation, so the overall pI of pH-PPI is at pH 4.5, where it carries no net charge. The salt-PPI, on the other hand, contained albumin proteins (Figure 1), which have an isoelectric point around pH 5.5–6 as explained earlier. Therefore, the overall pI of salt-PPI is likely higher than pH 4.5. Proteins carry more charge as they are farther from their isoelectric point, thus the pH-PPI had higher net negative charge than the salt-PPI.

The surface charge of SU-pH PPI was significantly lower than that of benchtop pH-PPI. This observation is most likely attributed to the partial denaturation incurred during SU production. Upon denaturation of globular proteins, uncharged hydrophobic groups get exposed, causing a reduction in the measured zeta potential. On the other hand, while SU-salt PPI was also partially denatured, it had slightly higher surface charge than the benchtop salt-PPI. Although statistically significant, this difference could not be explained by the partial unfolding of the protein. Difference in ash content and the protein subunits that get involved in polymerization could be behind this observed difference. Polymerization, as noted by SDS-PAGE, involved partially the 25 kDa albumin subunit (Figure 1A, lane 3 vs. 5). Albumins, as mentioned before, would have less surface charge at pH 7 compared to globulins, thus polymerization involving albumin proteins may lead to the perceived higher net surface charge of SU-salt PPI compared to benchtop salt-PPI.

cPPI had the lowest surface charge compared to the other PPI samples, except for salt-PPI. cPPI was completely denatured (Table 3). The unraveling of the protein structure resulted in exposure of hydrophobic groups and consequently extensive protein aggregation (Figure 1). Although cSPI was similarly denatured and polymerized, it maintained higher net negative surface charge than most other proteins.

### 3.7. Protein Functionality as Impacted by the Extraction Method and Scale

Protein solubility of the different PPI samples was evaluated against not only commercial PPI and SPI, but also against a commercial WPI (cWPI). WPI has been considered the “gold standard” for use in high protein beverages, as it is highly soluble (at 5–10% protein) even post-thermal treatment, at both neutral and acidic pH [23]. WPI maintains high solubility over a wide range of pH since it has relatively low surface hydrophobicity (RFI of 4051) compared to PPI and SPI samples (Table 3). Accordingly, all PPI samples and reference cPPI and cSPI had significantly lower solubility than cWPI at pH 7, and more so at pH 3.4 (Table 4). Solubility was measured at both pH 7 and pH 3.4 to indicate potential use of pea protein in neutral and acidic high protein beverages as a replacement for WPI.

At pH 7, the pH-extracted PPI samples had significantly higher protein solubility than the salt-extracted PPI samples, which could partially be attributed to their relatively higher surface charge (Table 3). The observed difference could also be attributed to the selective solubilization of proteins at pH 7.5 during the pH-extraction method. During salt extraction, the solubilized proteins would not necessarily have selective solubility at a pH close to 7. On the other hand, partial denaturation of the pH-extracted PPI imparted by SU production did not majorly impact protein solubility at pH 7 under both not-heated and heated conditions. On the other hand, the solubility of SU-salt PPI at pH 7 was significantly lower than that of salt-PPI when not-heated yet was comparable when heated. This observation could be attributed to the combined effect of surface charge and denaturation. The thermal treatment caused partial denaturation of the major proteins in SU-salt PPI, and significantly increased the surface hydrophobicity compared to that of the benchtop salt-PPI (Table 3). However, heating the protein solutions at 80 °C for 30 min most likely resulted in partial denaturation of the proteins in the benchtop salt-PPI, but did not cause further changes to the proteins in SU-salt PPI.

At pH 3.4, salt-PPI, when not-heated and when heated, had significantly higher solubility than pH-PPI (Table 4). Since pH-extraction selected for proteins with an isoelectric point of 4.5, pH-PPI demonstrated comparatively lower solubility at pH 3.4 than salt-PPI. The lower net negative charge of salt-PPI at pH 7 compared to that of pH-PPI (Table 3) indicated that the pI of salt-PPI was higher than 4.5, potentially due to higher albumin content as explained earlier. Thus, the salt-PPI would carry higher net positive charge at pH 3.4 than pH-PPI. Upon partial protein denaturation in the SU samples, the potential difference in net positive charge between the salt extracted and pH extracted samples had no apparent impact on protein solubility. However, partial denaturation did result in significantly lower protein solubility of SU-salt PPI compared to salt-PPI, with no apparent influence of another factor. The ash contents of the SU-salt PPI and salt-PPI were not statistically different (1.66% ash for SU-salt PPI, 1.56% ash for benchtop salt-PPI), thus could not have contributed to differences in solubility. At pH 3.4, the net (positive) surface charge would be lower than the net (negative) surface charge at pH 7, resulting in potentially less repulsion among the partially denatured proteins, allowing for hydrophobic interactions and subsequent aggregation. On the other hand, The SU-pH PPI had significantly higher solubility at pH 3.4 when not-heated than the benchtop pH-PPI. This observation could be attributed to their different ash contents (4.96% ash for benchtop pH-PPI, 2.94% ash for SU-pH PPI; *p* < 0.05). At low levels, salt can help increase protein solubilization by “salting in” the proteins. However, after a certain point, salt content may be high enough to shield surface charge on the protein, thereby decreasing solubility [13,52]. This effect was not seen at pH 7, likely because the proteins carried higher charge further from their isoelectric point, so the salt content might not have had a major impact. However, at a pH closer to pH 4.5, the proteins carried less net charged, so the effects of shielding by salt ions were relatively more pronounced.

Regardless of extraction method or scale, PPI samples had comparable solubility to cSPI and superior solubility to cPPI at pH 7 and had superior solubility to both cSPI and cPPI at pH 3.4 (Table 4). Shand [32] similarly observed lower solubility of commercial PPI and SPI compared to native PPI and SPI. The authors attributed this observation to the denaturation and polymerization upon spray drying [32]. However, the SU PPI samples produced in the current study were also spray dried, indicating that the poor solubility of cPPI and cSPI were caused by harsh extraction/processing conditions other than spray drying. The high alkalinity (pH 9–11) used in different studies [7,9,10,11] and in industry to enhance extraction yield, is the likely cause of reduced solubility. These findings confirm that SU production of PPI under controlled conditions (e.g., lower pH coupled with double solubilization) will contribute to relatively high and acceptable solubility compared to current industrial processes.

While preserving the structural integrity of pea protein through controlled extraction conditions had a pronounced impact on solubility, its effect on gelation was modest. Gelation is impacted greatly by the inherent protein profile and characteristics. Due to relatively higher legumin (glycinin) to vicilin (β-conglycinin) ratio [1,32,53] in soy protein compared to pea protein, cSPI formed the strongest gel compared to all PPI samples (Table 4). In contrast to vicilin, legumin proteins contain cysteine residues that can form inter- and intramolecular linkages, contributing to a uniform structure and strength to a gel matrix [54]. Accordingly, PPI gels are believed to be primarily formed through hydrophobic interactions among vicilin proteins, with minor disulfide stabilization by the small amount of legumin present [32,53]. Due to this difference in the inherent protein profile, cSPI formed a relatively strong gel at 15% protein, while 20% protein concentration was needed for the PPI samples to form cohesive gels. Even at 20% protein concentration, the PPI gels formed were significantly weaker than the cSPI gel (at 15% protein). In fact, cPPI formed a very weak get that fell apart before a gel rupture force can be recorded. cPPI had low surface charge, was completely denatured, extensively aggregated, and irreversibly polymerized. It also had very low solubility at pH 7 compared to the other samples. Accordingly, heating cPPI caused further, yet random, aggregation of the proteins in the form of coagulum that was unable to entrap water. Taherian [20] similarly reported that commercial PPI did not form a gel at 20% protein, an observation they attributed to poor solubility due to denaturation and polymerization that occurred during protein extraction.

Both the benchtop and SU PPIs were superior to cPPI in their ability to form a gel, as they were less denatured during extraction and processing. The benchtop salt-PPI approached cSPI in terms of gel strength and had significantly the highest gel strength among the PPI samples (Table 4). Salt-PPI had slightly lower surface charge than the other PPI samples, and considerably lower surface hydrophobicity (Table 3), indicating a potentially more suitable hydrophilic to hydrophobic balance for a balanced protein-water and protein–protein interactions. Protein–protein interactions are required for a gel matrix to form. However, the presence of pre-formed large polymers/aggregates among denatured protein may have a negative effect on gel strength [55]. On the other hand, proteins that are less denatured and not polymerized will further denature during heating and then proceed to form soluble aggregate, leading to the formation of an ordered protein network [56,57]. The SU-pH and SU-salt PPI samples were partially denatured and aggregated (Table 3 and Figure 1), and the pH-PPI showed slight aggregation. While SU-pH PPI, SU-salt PPI, and pH-PPI still formed a gel, the gels likely formed through random associations compared to that of the salt-PPI, which was the least denatured and did not have any signs of aggregation (Figure 1 and Table 3).

cSPI also outperformed all PPI samples in most of the emulsification properties (Table 4), attributed to its moderate surface hydrophobicity and relatively high surface charge, resulting in a superior hydrophilic to lipophilic balance (HLB) compared to the PPI samples. All PPI samples did not form emulsion at 1% protein, thus had to be tested at 2% protein instead. The poor solubility, low surface charge, high surface hydrophobicity, and excessive polymerization inhibited cPPI from effectively interacting with either phase, resulting in the least EC among the samples. On the other hand, benchtop and SU PPIs had comparable EC values that were significantly higher than that of cPPI (Table 4).

Compared to cSPI, benchtop pH-SPI had similar EAI, and salt-PPI had significantly higher EAI. This observation indicated that salt-SPI, compare to all other samples, more readily migrated to the water/oil interface, and oriented their hydrophobic residues to the oil phase and hydrophilic residues to the water. Proteins that have flexible structures and good solubility have relatively high EAI [26,58,59]. Salt-PPI was the least denatured and polymerized among the samples, potentially contributing to ease of migration to the interface.

While SU PPI samples had significantly lower EAI than cSPI, they had comparable ES. ES is related to the thickness of the protein film at the interface and the charge on the surface of the oil droplets needed to cause repulsion among emulsified oil droplets, preventing coalescence. Results indicated that SU PPI samples were able to form a relatively thick film at the interface, leading to good ES, although they had lower EC and EAI than cSPI.

Of all protein isolates tested, cPPI had the highest ES but the lowest EAI (Table 4). As cPPI was highly denatured and aggregated, they are relatively less flexible to easily migrate to the water/oil interface. The poor solubility and high extent of denaturation and aggregation in cPPI would cause the protein to interact with each other rather than efficiently migrating to the water/oil interface, thus increasing the viscosity of the continuous phase, and contributing to the stability of the formed oil droplets [60].

## 4. Conclusions

Pea protein extraction was evaluated, with a focus on selecting conditions that are scalable and can preserve the structural integrity of the protein. Both pH- and salt- extractions under controlled conditions resulted in protein purity and yield comparable or superior to what has been reported previously for pea protein extractions. Particularly, extraction at close to neutral pH coupled with double solubilization, not only resulted in good protein yield and purity, but also resulted in preserved structure and superior functionality to cPPI. Results of this work, confirmed for the first time that double solubilization at mild pH can replace single solubilization at high alkalinity, and achieve similar yield, while preserving structural integrity. While there were some structural and functional differences in salt-PPI compared to pH-PPI, scaling up the production eliminated such differences. Additionally, this study confirmed, for the first time in pea, the scalability of the benchtop salt extraction coupled with membrane filtration. Scaling up under mild and controlled conditions resulted in partial denaturation and low degree of polymerization compared to cPPI. Both SU PPI samples had superior functionality compared to cPPI, produced under harsh pH-extraction conditions. These results provided valuable insights in scalability of specific benchtop parameters, which will guide industrial production of functional pea protein ingredient. In comparison to cSPI, both SU PPI had comparable solubility to cSPI at pH 7 and superior solubility at pH 3.4, making them suitable for acidic protein beverages; however, both SU PPI had inferior gelation and emulsification properties. In order to enhance the functionality of pea protein and expand their utilization, structural modification through physical, enzymatic, and chemical (such as Maillard glycation) approaches can be further explored. Given the inherent protein composition and distribution of the different pea protein fractions, selective breeding with targeted protein phenotyping could be another plausible approach to further enhance the prospects of pea protein.

## Figures and Tables

**Table 1:** Percent protein purity and distribution in the PPI, pellet, and supernatant fractions from pH and salt extractions under different conditions, as well as ash content (%) of each PPI sample.

pH Extractions											
Extraction Treatment					pH-PPI			Discarded Pellet ^1^		Discarded Supernatant ^2^	
Solubilization pH	Precipitation pH	Solubilization Duration (h)	Number of Solubilizations	Dialysis of PPI	Protein Purity (%)	Protein Yield ^3^ (%)	Ash (%)	Protein Purity (%)	Protein Residue ^4^ (%)	Protein Purity (%)	Protein Lost ^5^ (%)
7	4.5	1	1	No	89.7 ^a6^	56.3 ^eB7^	5.11 ^a^	8.08 ^aA^	22.1 ^aA^	29.5 ^b^	18.7 ^d^
8	4.5	1	1	No	86.5 ^ab^	60.9 ^cA^	5.02 ^a^	6.76 ^abB^	18.9 ^aB^	29.5 ^b^	19.7 ^cd^
7.5	4.5	1	1	No	88.3 ^ab^	58.1 ^cdeB^	5.03 ^a^	6.31 ^abcB^	18.2 ^aB^	28.7 ^bc^	19.3 ^cd^
7.5	5	1	1	No	89.4 ^a^	57.5 ^de^	4.24 ^b^8^	5.82 ^bc^	17.1 ^a^	31.5 ^a^^	23.4 ^a^^
7.5	4.5	2	1	No	85.0 ^ab^	60.5 ^cd^	5.07 ^a^	5.66 ^bc^	16.6 ^ab^	27.7 ^bc^	20.1 ^c^
7.5	4.5	1	2	No	84.5 ^b^	69.9 ^a☨9^	5.19 ^a^	3.00 ^d☨^	8.11 ^c☨^	27.4 ^c☨^	21.8 ^b☨^
7.5	4.5	1	2	Yes	87.6 ^ab^	64.7 ^b^*^10^	4.96 ^a^	4.30 ^cd^	11.2 ^bc^	28.0 ^bc^	21.6 ^b^
Salt Extractions											
Purification Treatment					Salt-PPI			Discarded Pellet			
Ultrafiltration		Dialysis of PPI			Protein Purity (%)	Protein Yield (%)	Ash (%)	Protein Purity (%)		Protein Residue (%)	
Yes		No			67.9 ^c^	76.1 ^a^	11.4 ^a^	7.97 ^a^		25.2 ^a^	
No		Yes			86.9 ^b^	69.7 ^b^	7.19 ^b^	7.98 ^a^		25.3 ^a^	
Yes		Yes			92.8 ^a^^*	72.0 ^ab^*	1.56 ^c^^*	7.70 ^a^		24.2 ^a^	

^1^ Pellet discarded after alkaline or salt solubilization; ^2^ Supernatant discarded after isoelectric precipitation; ^3^ Protein yield (%) represents the amount of protein extracted relative to the total amount of protein in the starting pea flour; ^4^ Protein residue (%) represents the amount of protein left in the discarded pellet relative to the total amount of protein in the starting pea flour; ^5^ Protein lost (%) represents the amount of protein lost to the discarded supernatant relative to the total amount of protein in the starting pea flour during pH extractions; ^6^ Means (*n* = 3) in each column with different lowercase letters indicate significant differences across extraction treatments; ^7^ Means with different capital letters indicate significant differences among different solubilization pHs; means with no capital letters indicate no significant differences, according to the Tukey–Kramer multiple means comparison test (*p* < 0.05); ^8^ A carrot (^^^) in pH extractions designates a significant difference among corresponding samples precipitated at pH 4.5 and 5, while a carrot (^^^) in salt extractions designates a significant difference among corresponding samples with and without ultrafiltration, as tested by the student’s two-sample unpaired *t*-test (*p* < 0.05); ^9^ A cross of Lorraine (☨) designates a significant difference among corresponding samples solubilized once or twice, as tested by the student’s two-sample unpaired *t*-test (*p* < 0.05); ^10^ An asterisk (*) designates a significant difference among a corresponding dialyzed and non-dialyzed sample, as tested by the student’s two-sample unpaired t-test (*p* < 0.05).

**Table 2:** Relative abundance of soluble aggregates and functional proteins present in cPPI reference, and PPI samples produced at bench scale and pilot scale.

Protein Fractions ^1^	Relative Abundance (%) ^2^				
	cPPI	pH-PPI	Salt-PPI	SU-pH PPI	SU-Salt PPI
Soluble aggregates (Association of legumin, vicilin and other protein fractions)	13.06 ^b3^	2.07 ^d^	2.55 ^d^	18.06 ^a^	9.91 ^c^
Legumin	*^4^	28.23 ^a^	25.41 ^a^	19.47 ^b^	19.67 ^b^
Convicilin	*	7.30 ^a^	7.23 ^a^	5.57 ^b^	5.18 ^b^
Vicilin	3.42 ^e^	10.44 ^b^	11.38 ^a^	6.84 ^c^	5.63 ^d^

^1^ Samples were dissolved in pH 7 phosphate buffer and analyzed by high-performance size exclusion chromatography (SE-HPLC); ^2^ Relative abundance (%) is the area of a specific peak divided by the total peak area for that sample; ^3^ Means (*n* = 3) in each row with different lowercase letters indicate significant differences according to the Tukey–Kramer multiple means comparison test (*p < 0.05*); ^4^ An asterisk (*) indicates that no peak was detected in this molecular weight range.

**Table 3:** Denaturation temperatures and enthalpy, surface hydrophobicity and surface charge of commercial protein references and the different PPI samples produced at bench scale and pilot scale.

Plant Protein Isolate	Denaturation Temperature and Enthalpy				Surface Hydrophobicity	Surface Charge
	Denaturation Temperature (Td, °C)	Enthalpy of Denaturation (ΔH, J g^−1^)	Denaturation Temperature (Td, °C)	Enthalpy of Denaturation (ΔH, J g^−1^)	RFI	mV
	Vicilin		Legumin			
cPPI	*^1^	*	*	*	12,718 ^ab^	−31.1 ^cd^
pH-PPI	83.3 ^b2^	6.21 ^a^	91.6 ^ab^	0.81 ^b^	9667 ^cd^	−40.2 ^a^
salt-PPI	88.5 ^a^	6.82 ^a^	93.5 ^a^	1.54 ^a^	7161 ^d^	−30.4 ^d^
SU-pH PPI	81.9 ^c^	3.69 ^b^	90.0 ^b^	0.52 ^b^	15,131 ^a^	−34.5 ^b^
SU-salt PPI	82.4 ^c^	4.15 ^b^	90.2 ^b^	0.47 ^b^	13,114 ^ab^	−32.7 ^bc^
	β-conglycinin		Glycinin			
cSPI	*	*	*	*	11,662 ^bc^	−41.1 ^a^

^1^ An asterisk (*) represents no peak of denaturation observed; ^2^ Means (*n* = 3) in each column with different lowercase letters indicate significant differences among samples, according to the Tukey–Kramer multiple means comparison test (*p* < 0.05).

**Table 4:** Solubility, gel strength and emulsification properties of commercial protein references and PPI samples produced at bench scale and pilot scale.

Protein Isolate	% Protein Solubility (5% Protein)				Gel Strength (15 or 20% Protein) ^1^	Emulsification Capacity (1 or 2% Protein) ^2^	EAI	ES
	*pH 7*		*pH 3.4*		N	mL Oil/g of Protein	m^2^/g Oil	Min
	Not-Heated	Heated at 80 °C	Not-Heated	Heated at 80 °C				
cWPI	99.7 ^a3^	99.7 ^a^	99.4 ^a^	100.0 ^a^	N/A ^4^	N/A	N/A	N/A
cSPI	79.7 ^d^	86.9 ^b^*^5^	23.7 ^f^	25.9 ^d^	17.3 ^a^	1085 ^a^	131.8 ^b^	32.5 ^bc^
cPPI	22.2 ^f^	39.0 ^d^*	8.9 ^g^	20.4 ^d*^	N/A	260 ^d^	88.7 ^c^	45.4 ^a^
pH-PPI	87.4 ^b^	85.7 ^b^	43.6 ^e^	65.4 ^c^*	6.1 ^c^	441 ^b^	149.1 ^b^	36.9 ^b^
salt-PPI	84.1 ^c^	72.0 ^c^*	88.7 ^b^	90.1 ^b^	14.6 ^b^	452 ^b^	197.4 ^a^	26.0 ^d^
SU-pH PPI	88.4 ^b^	86.8 ^b^	68.4 ^c^	61.6 ^c^	7.5 ^c^	359 ^c^	95.1 ^c^	36.7 ^bc^
SU-salt PPI	70.4 ^e^	71.5 ^c^	64.0 ^d^	68.6 ^c^	5.7 ^c^	403 ^bc^	72.4 ^c^	30.8 ^cd^

^1^ SPI gel samples were prepared at 15% protein (*w*/*v*) and heated for 10 min at 95 °C, while PPI gels were prepared at 20% protein (*w*/*v*) and heated for 20 min at 95 °C; ^2^ SPI EC samples were prepared at 1% protein (*w*/*v*), while PPI EC samples were prepared at 2% protein (*w*/*v*); ^3^ Means (*n* = 3) in each column with different lowercase letters indicate significant differences among samples, according to the Tukey–Kramer multiple means comparison test (*p* < 0.05); ^4^ N/A: not available; ^5^ An asterisk (*) indicates a significant difference between a not-heated and heated sample (*p* < 0.05).

## Data Availability

Data available upon reasonable request from the corresponding author.

## Abbreviations

cPPI: commercial pea protein isolate; cSPI, commercial soy protein isolate; cWPI, commercial whey protein isolate; DSC, differential scanning calorimetry; EAI, emulsification activity index; EC, emulsification capacity; ES, emulsification stability; pH-PPI, pH extracted pea protein isolate; salt-PPI, salt extracted pea protein isolate; SDS-PAGE, sodium dodecyl sulfate-polyacrylamide gel electrophoresis; SE-HPLC, size-exclusion high-performance liquid chromatography; SU, scaled-up; SU-pH PPI, scaled-up pH extracted pea protein isolate; SU-salt PPI, scaled-up salt extracted pea protein isolate. UF, ultrafiltration; WPI, whey protein isolate.

## References

[1] Tulbek M.C., Lam R.S.H., Wang Y., Asavajaru P., Lam A., Nadathur S.R., Wanasundara J.P.D., Scanlin L. (2017). Chapter 9—Pea: A Sustainable Vegetable Protein Crop. Sustainable Protein Sources.

[2] Chang C., Tu S., Ghosh S., Nickerson M. (2015). Effect of pH on the inter-relationships between the physicochemical, interfacial and emulsifying properties for pea, soy, lentil and canola protein isolates. Food Res. Int..

[3] Zhao H., Shen C., Wu Z., Zhang Z., Xu C. (2020). Comparison of wheat, soybean, rice, and pea protein properties for effective applications in food products. J. Food Biochem..

[4] Bu F., Nayak G., Bruggeman P., Annor G., Ismail B.P. (2022). Impact of plasma reactive species on the structure and functionality of pea protein isolate. Food Chem..

[5] Adebiyi A.P., Aluko R.E. (2011). Functional properties of protein fractions obtained from commercial yellow field pea (*Pisum sativum* L.) seed protein isolate. Food Chem..

[6] Arntfield S.D., Maskus H.D., Phillips G.O., Williams P.A. (2011). 9-Peas and other legume proteins. Handbook of Food Proteins.

[7] Cui L., Bandillo N., Wang Y., Ohm J.-B., Chen B., Rao J. (2020). Functionality and structure of yellow pea protein isolate as affected by cultivars and extraction pH. Food Hydrocolloid.

[8] Pelgrom P.J. (2015). Dry Fractionation for Sustainable Production of Plant Protein Concentrates.

[9] Gao Z., Shen P., Lan Y., Cui L., Ohm J.-B., Chen B., Rao J. (2020). Effect of alkaline extraction pH on structure properties, solubility, and beany flavor of yellow pea protein isolate. Food Res. Int..

[10] Tanger C., Engel J., Kulozik U. (2020). Influence of extraction conditions on the conformational alteration of pea protein extracted from pea flour. Food Hydrocolloid.

[11] Karaca A.C., Low N., Nickerson M. (2011). Emulsifying properties of chickpea, faba bean, lentil and pea proteins produced by isoelectric precipitation and salt extraction. Food Res. Int..

[12] Murray E., Myers C., Barker L., Maurice T. (1981). Functional attributes of proteins—A noncovalent approach to processing and utilizing plant proteins. Util. Protein Resour..

[13] Duong-Ly K.C., Gabelli S.B. (2014). Salting out of proteins using ammonium sulfate precipitation. Methods in Enzymology.

[14] Xu H.-N., Liu Y., Zhang L. (2015). Salting-out and salting-in: Competitive effects of salt on the aggregation behavior of soy protein particles and their emulsifying properties. Soft Matter.

[15] Deak N., Murphy P., Johnson L. (2006). Effects of NaCl concentration on salting-in and dilution during salting-out on soy protein fractionation. J. Food Sci..

[16] Hayati Zeidanloo M., Ahmadzadeh Ghavidel R., Ghiafeh Davoodi M., Arianfar A. (2019). Functional properties of Grass pea protein concentrates prepared using various precipitation methods. J. Food Sci. Technol..

[17] Stone A.K., Karalash A., Tyler R.T., Warkentin T.D., Nickerson M.T. (2015). Functional attributes of pea protein isolates prepared using different extraction methods and cultivars. Food Res. Int..

[18] Gao L.L., Nguyen K.D., Utioh A.C. (2001). Pilot scale recovery of proteins from a pea whey discharge by ultrafiltration. LWT-Food Sci. Technol..

[19] Tian S., Kyle W.S., Small D.M. (1999). Pilot scale isolation of proteins from field peas (*Pisum sativum* L.) for use as food ingredients. Int. J. Food Sci. Technol..

[20] Taherian A.R., Mondor M., Labranche J., Drolet H., Ippersiel D., Lamarche F. (2011). Comparative study of functional properties of commercial and membrane processed yellow pea protein isolates. Food Res. Int..

[21] Laemmli U.K. (1970). Cleavage of structural proteins during the assembly of the head of bacteriophage T4. Nature.

[22] Boyle C., Hansen L., Hinnenkamp C., Ismail B.P. (2018). Emerging camelina protein: Extraction, modification, and structural/functional characterization. J. Am. Oil Chem. Soc..

[23] Wang Q., Ismail B. (2012). Effect of Maillard-induced glycosylation on the nutritional quality, solubility, thermal stability and molecular configuration of whey protein. Int. Dairy J..

[24] Hinnenkamp C., Ismail B.P. (2021). Enhancing emulsion stability: The synergistic effect of combining Procream and partially hydrolyzed whey protein. Int. Dairy J..

[25] Hoang H.D. (2012). Evaluation of Pea Protein and Modified Pea Protein as Egg Replacers. Ph.D. Thesis.

[26] Feyzi S., Milani E., Golimovahhed Q.A. (2018). Grass Pea (*Lathyrus sativus* L.) Protein Isolate: The Effect of Extraction Optimization and Drying Methods on the Structure and Functional Properties. Food Hydrocolloid.

[27] Reinkensmeier A., Bußler S., Schlüter O., Rohn S., Rawel H.M. (2015). Characterization of individual proteins in pea protein isolates and air classified samples. Food Res. Int..

[28] Lee H., Htoon A., Uthayakumaran S., Paterson J. (2007). Chemical and functional quality of protein isolated from alkaline extraction of Australian lentil cultivars: Matilda and Digger. Food Chem..

[29] Barac M., Cabrilo S., Stanojevic S., Pesic M., Pavlicevic M., Zlatkovic B., Jankovic M. (2012). Functional properties of protein hydrolysates from pea (*Pisum sativum*, L.) seeds. Int. J. Food Sci. Technol..

[30] Boye J., Aksay S., Roufik S., Ribéreau S., Mondor M., Farnworth E., Rajamohamed S. (2010). Comparison of the functional properties of pea, chickpea and lentil protein concentrates processed using ultrafiltration and isoelectric precipitation techniques. Food Res. Int..

[31] O'Kane F.E., Happe R.P., Vereijken J.M., Gruppen H., Van Boekel M.A.J.S. (2004). Characterization of Pea Vicilin. 1. Denoting Convicilin as the α-Subunit of thePisumVicilin Family. J. Agric. Food Chem..

[32] Shand P.J., Ya H., Pietrasik Z., Wanasundara P.K.J.P.D. (2007). Physicochemical and textural properties of heat-induced pea protein isolate gels. Food Chem..

[33] Sumner A., Nielsen M., Youngs C. (1981). Production and evaluation of pea protein isolate. J. Food Sci..

[34] Gueguen J., Cerletti P. (1994). Proteins of some legume seeds: Soybean, pea, fababean and lupin. New and Developing Sources of Food Proteins.

[35] Taherian A.R., Mondor M., Lamarche F. (2012). Enhancing nutritional values and functional properties of yellow pea protein via membrane processing. Peas Cultiv. Var. Nutr. Uses.

[36] Lu Z.X., He J.F., Zhang Y.C., Bing D.J. (2019). Composition, physicochemical properties of pea protein and its application in functional foods. Crit. Rev. Food Sci. Nutr..

[37] Owusu-Ansah Y.J., McCurdy S.M. (1991). Pea proteins: A review of chemistry, technology of production, and utilization. Food Rev. Int..

[38] Hensley D., LAWHON J.T. (1979). Economic evaluation of soy isolate production by a membrane isolation process. Food Technol..

[39] Tzitzikas E.N., Vincken J.-P., De Groot J., Gruppen H., Visser R.G.F. (2006). Genetic Variation in Pea Seed Globulin Composition. J. Agric. Food Chem..

[40] Mession J.-L., Chihi M.L., Sok N., Saurel R. (2015). Effect of globular pea proteins fractionation on their heat-induced aggregation and acid cold-set gelation. Food Hydrocolloid.

[41] Croy R.R., Hoque M.S., Gatehouse J.A., Boulter D. (1984). The major albumin proteins from pea (*Pisum sativum* L.) Purification and some properties. Biochem. J..

[42] Swanson B.G. (1990). Pea and lentil protein extraction and functionality. J. Am. Oil Chem. Soc..

[43] Makri E., Papalamprou E., Doxastakis G. (2005). Study of functional properties of seed storage proteins from indigenous European legume crops (lupin, pea, broad bean) in admixture with polysaccharides. Food Hydrocolloid.

[44] Mession J.-L., Sok N., Assifaoui A., Saurel R.m. (2013). Thermal Denaturation of Pea Globulins (*Pisum sativum* L.) Molecular Interactions Leading to Heat-Induced Protein Aggregation. J. Agric. Food Chem..

[45] Phillips G.O., Williams P.A. (2011). Handbook of Food Proteins.

[46] Damodaran S., Parkin K.L. (2017). Amino acids, peptides, and proteins. Fennema’s Food Chemistry.

[47] Friedman M. (1999). Chemistry, biochemistry, nutrition, and microbiology of lysinoalanine, lanthionine, and histidinoalanine in food and other proteins. J. Agric. Food Chem..

[48] Lee K., Ryu H., Rhee K. (2003). Protein solubility characteristics of commercial soy protein products. J. Am. Oil Chem. Soc..

[49] Foegeding E.A., Davis J.P. (2011). Food protein functionality: A comprehensive approach. Food Hydrocolloid.

[50] Rickert D., Johnson L., Murphy P. (2004). Functional Properties of Improved Glycinin and β-nglycinin Fractions. J. Food Sci..

[51] Lampart-Szczapa E. (2001). Legume and oilseed proteins. Chem. Funct. Prop. Food Proteins.

[52] Zhou H.X. (2005). Interactions of macromolecules with salt ions: An electrostatic theory for the Hofmeister effect. Proteins Struct. Funct. Bioinform..

[53] Sun X.D., Arntfield S.D. (2011). Gelation properties of salt-extracted pea protein isolate induced by heat treatment: Effect of heating and cooling rate. Food Chem..

[54] Wolf W.J. (1970). Soybean proteins. Their functional, chemical, and physical properties. J. Agric. Food Chem..

[55] Sun X.D., Arntfield S.D. (2010). Gelation properties of salt-extracted pea protein induced by heat treatment. Food Res. Int..

[56] Tombs M. (1974). Gelation of globular proteins. Faraday Discuss. Chem. Soc..

[57] Hermansson A.-M. (1979). Aggregation and denaturation involved in gel formation. Functionality and Protein Structure.

[58] Nakai S. (1983). Structure-function relationships of food proteins: With an emphasis on the importance of protein hydrophobicity. J. Agric. Food Chem..

[59] Barac M., Cabrilo S., Pesic M., Stanojevic S., Zilic S., Macej O., Ristic N. (2010). Profile and Functional Properties of Seed Proteins from Six Pea (*Pisum sativum*) Genotypes. Int. J. Mol. Sci..

[60] Nour A.H. (2018). Emulsion types, stability mechanisms and rheology: A review. Int. J. Innov. Res. Sci. Stud. (IJIRSS).

